# Changes in sexual activities, function, and satisfaction during the COVID-19 pandemic era: a systematic review and meta-analysis

**DOI:** 10.1093/sexmed/qfad005

**Published:** 2023-03-24

**Authors:** Kowsar Qaderi, Mansoureh Yazdkhasti, Sanaz Zangeneh, Bahar Morshed Behbahani, Mehri Kalhor, Ahmadreza Shamsabadi, Younes Jesmani, Solmaz Norouzi, Mehrnaz Kajbafvala, Rasa Khodavirdilou, Nahid Rahmani, Masoumeh Namadian, Sajjad Ghane Ezabadi, Ibrahim Alkatout, Esmaeil Mehraeen, Dara Rasoal

**Affiliations:** Midwifery Department, School of Nursing and Midwifery, Kermanshah University of Medical Sciences, Kermanshah, Iran; Reproductive Health Department, Social Determinants of Health Research Center, Alborz University of Medical Sciences, Karaj, Iran; Midwifery and Reproductive Health Department, Student Research Committee, School of Nursing and Midwifery, Isfahan University of Medical Sciences, Isfahan, Iran; Department of Midwifery, School of Nursing and Midwifery, Shiraz University of Medical Sciences, Shiraz, Iran; Kowsar Hospital, Qazvin University of Medical Sciences, Tehran, Iran; Department of Health Information Technology, Esfarayen Faculty of Medical Science, Esfarayen, Iran; Medicine School, Kermanshah University of Medical Sciences, Kermanshah, Iran; Department of Statistics and Epidemiology, School of Medicine, Zanjan University of Medical Sciences, Zanjan, Iran; Rehabilitation Research Center, Department of Physiotherapy, School of Rehabilitation Sciences, Iran University of Medical Sciences, Tehran, Iran; Department of Reproductive Biology, Faculty of Advanced Medical Sciences, Tabriz University of Medical Sciences, Tabriz, Iran; Pediatric Neurorehabilitation Research Center, Department of Physiotherapy, University of Social Welfare and Rehabilitation Sciences, Tehran, Iran; Social Determinants of Health Research Center, Zanjan University of Medical Sciences, Zanhan, Iran; School of Medicine, Tehran University of Medical Sciences, Tehran, Iran; Klinik für Gynäkologie und Geburtshilfe, Universitätsklinikum Schleswig-Holstein, Campus Kiel, Leitender Oberarzt Leiter der Kiel School of Gynaecological Endoscopy, Kiel, Germany; Department of Health Information Technology, Khalkhal University of Medical Sciences, Khalkhal, Iran; Institution of Health and Social Welfare, Dalarna University, Falun 6631145632, Sweden

**Keywords:** COVID-19, SARS-CoV-2, systematic review, female sexual dysfunction, sexual behavior, general population

## Abstract

**Introduction:**

Little is known about the impact of the coronavirus on sexual behavior, function, and satisfaction.

**Aim:**

The aim of the present study was to systematically review people’s sexual function and behaviors and their changes in sexual activities during the COVID-19 pandemic.

**Methods:**

Comprehensive searches in PubMed, Web of Science, and Scopus were conducted with keywords in accordance with MeSH terms: COVID-19, SARS-CoV-2, coronavirus, sexual health, sexual function, sexual dysfunctions, sexuality, sexual orientation, sexual activities, and premarital sex. Two reviewers independently assessed full-text articles according to predefined criteria: original design, English studies, and investigating either the general population or sexual minorities.

**Results:**

Risk of bias in the studies was assessed by the Newcastle-Ottawa Scale, and data were pooled via random effects meta-analyses. We utilized the standardized mean difference to evaluate the effects of the COVID-19 pandemic on sexual activity, functioning, and satisfaction. We included 19 studies in the analysis and 11 studies in the meta-analysis, with a sample size of 12 350. To investigate sexual activity changes, a sample size of 8838 was entered into the subgroup analysis, which showed a significant decrease in both genders (5821 women, *P* < .033; 3017 men, *P* < .008). A subgroup meta-analysis showed that the sexual function of men and women during the COVID-19 pandemic significantly declined (3974 women, *P* < .001; 1427 men, *P* < .001). Sexual desire and arousal decreased in both genders, though mainly in women. In investigating sexual satisfaction changes during the COVID-19 pandemic, a meta-analysis with a sample size of 2711 showed a significant decrease (*P* < .001). The most indicative changes in sexual behaviors during the pandemic were the increase in masturbating and usage of sex toys. Greater COVID-19 knowledge was associated with lower masturbation, oral sex, and vaginal sex. The more protective behaviors were associated with less hugging, kissing, cuddling, genital touching, watching porn with a partner, and vaginal sex.

**Conclusion:**

The COVID-19 pandemic led to increased challenges and changes for individuals’ sexual behaviors. Efforts for preventive strategies should therefore be concentrated between pandemics, while ensuring that there is information available to the population during a pandemic for help in times of psychological distress or crisis.

## Introduction

The COVID-19 pandemic, as a major health issue, has had important effects on well-being,^1^^,^^2^ since the World Health Organization documented it as a global pandemic.^3^ Worldwide, as of August 18, 2021, approximately 208 million infections (confirmed cases) and 4.37 million deaths have been reported to the World Health Organization.^3^^,^^4^ This pandemic has had a significant adverse impact on people’s psychological, emotional, and socioeconomic status.^5^^,^^6^ Anxiety, stress, fear of contagion, fear of death, physical inactivity and weight gain, changes in sleep patterns, and fear of job loss were the most common psychosocial consequences of the COVID-19 pandemic.^7^ Also, societies have experienced social distancing, unemployment, professions being shut down, and lower income.^8^ Isolation has changed individuals’ lifestyles and habits. People may have experienced the loss of relatives and separation from family or partners.^9^ This confinement has decreased people’s health care seeking, and the tendency of people to have sex seems to have changed.^10^^,^^11^ Previous studies showed that sexual behavior was considered to be risky during the quarantine and could result in sexual dysfunction and dissatisfaction, unwanted pregnancies, several sexually transmitted infections, and declining quality of life.^12^^,^^13^

Several studies have been conducted on the effect of COVID-19 on sexual behavior in the general population.^14^^,^^15^ The study conducted by Baran and Aykac in 2021 revealed that Turkish men had less sex, with lower quality, due to the fear of COVID-19 transmission.^16^ Another study reported that most people experienced unprotected sex and that the prevalence of early-phase syphilis increased.^17^ To date, there are few data about the impact of the coronavirus on people’s sexual lives, and there are some gaps and conflicting information about the effect of COVID-19 on sexual activities, function, and satisfaction.^18^ The study was designed to systematically review sexual function, activity, and satisfaction during the COVID-19 pandemic and to answer these questions:

- Was there a change in sexual activities during the COVID-19 pandemic as compared with prepandemic?
- Was there any changes in sexual functions in males and females during the COVID-19 pandemic?
- Were there gender differences in sexual behavior before and after the pandemic?
- Was there a change in sexual satisfaction during the COVID-19 pandemic?

## Methods

This systematic review was conducted in accordance with the PRISMA guidelines.^19^ A protocol for this study was registered in the PROSPERO registry for systematic reviews (CRD42021275387). We looked up the keywords of the study in the databases.

### Data sources

A literature search was conducted via Web of Science, Scopus, and PubMed. Additionally, we performed manual searches using the reference lists of retrieved studies.

### Search strategy

The search strategy of this review was organized in collaboration with 3 members of the author group. The complete search strategy is shown in Supplementary File 1. Key search terms included the following:

*A:* COVID-19 OR SARS-CoV-2 OR COVID OR “coronavirus”
*B:* “sexual health” OR “sexual behavior” OR “sexual dysfunctions” OR sexuality OR “sexual orientation” OR “sexual activities” OR “premarital sex”
*C:* A AND B.

### Eligibility criteria

We included the original articles related to our research question from December 2019, the beginning of the pandemic, until August 16, 2021. We included studies conducted on either the general population or sexual minorities.

The exclusion criteria were as follows:

- Nonoriginal studies: reviews, commentaries, letters to editor, opinions, or any studies with no original data
- Ongoing projects (eg, protocol articles)
- Non–full texts, conference abstracts, abstract papers, and short communications
- Non-English language
- Studies not related to the COVID-19 pandemic

### Data screening

EndNote software was utilized to organize articles identified in the review, and duplicate records of the same reports were removed. Two authors independently screened titles and abstracts against the inclusion and exclusion criteria.

### Data extraction

We extracted the following data from each study: (1) the first author’s name, the publication year, the location of the study, the study design, the number of participants, and the mean age of participants and (2) the results of sexual health status and sexual behavior. Three authors independently extracted data once studies were selected. To exclude any possible duplications, the selected articles were surveyed by the first author once more.

### Quality assessment

Quality assessment of articles in this study was based on the checklist of the Newcastle-Ottawa Scale. The quality assessment score of publications ranged from 6 to 8 and is shown in Table 1. Two experienced authors investigated the quality of the articles; any disagreement between them regarding the selection or deletion of articles, due to the lack of proper quality, was resolved by a third author.

**Table 1:** Impact of COVID-19 pandemic on sexual function and behaviors.

**Study** ^**a**^	**No.**	**Sex, No.**	**Age, y, mean ± SD (range)**	**Sexual function**	**Sexual activity** ^**b**^	**Sexual desire**	**Sexual satisfaction**	**Other findings**	**NOS**
Feng,^22^ China	284	Men: 134 Women: 150 Heterosexual	Men: 27.35 ± 4.23 Women: 27.04 ± 4.72 18-44	No. of sexual partners Reduced: 17.6% Unchanged: 78.5% Increase: 3.9% Quality of sexual life Bad: 17.6% General: 48.9% Good: 33.5%	Reduced 43.3% Unchanged 41.2% Increased 15.5%	Reduced: 25% Unchanged: 56% Increase: 19%	Reduced: 21.5% Unchanged: 46.1% Increased: 32.4%	Family function Severe dysfunction: 6.7% Moderate dysfunction: 40.5% Good function: 52.8 %	6
Fuchs,^25^ Poland	764	Women: 764 Heterosexual	25.1 ± 4.3 (18-40)	Arousal: decrease (*P* < .001) Lubrication: decrease (*P* < .001) Orgasm: decrease (*P* < .001) Pain: decrease (*P* < .001) Total FSFI: decrease (*P* < .001) Sexual dysfunction (FSFI <26) Increased (15.3% to 34.3%; *P* < .001)	Decrease (*P* < .001)	Decrease from 4.5 to 4.2 (*P* < .001)	Decrease (*P* < .001)	Women who do not work have lower FSFI scores than women who work.	7
Karagöz,^23^ Turkey	245	Men: 148 Women: 97 Heterosexual	Total: 35.9 ± 6.9 Men: 34.7 ± 6.67 Women: 36.7 ± 7.1	Erectile function: decrease (*P* < .001) Orgasmic function: decrease (*P* = .014) FSFI: decrease (*P* < .027) Arousal: decrease (*P* < .103) Lubrication: decrease (*P* < .034) Pain: decrease (*P* < .065) Increased masturbation, video, etc: 9.4% (female, 4.1%; male, 12.8%) New onset of single sexual approach: 4.1% (female, 5.2%; male, 3.4%) FSFI <26: increase (45.4% to 52.6%; *P* < .001)	Sexual intercourse per week ≤1 increased 2 decrease 2-5 decrease ≥5 Women: decreased Men: increased (*P* < .001 for all)	Decrease (*P* = .173)	Decrease (*P* < .001)	Taking precautions during intercourse Total: 9.8% Female: 14.4% Male: 6.8%	6
Baran,^16^ Turkey	536	Men: 536 Heterosexual	38.6 ± 10.3	IIEF: reduced (*P* = .015)	Reduced (*P* < .001)				6
Bourne,^15^ Jamaica	458	Men: 155 Women: 303 Heterosexual	18-20: 18.8% 21-30: 54.3% 31-40: 16.9% 41-60: 8.5% ≥61: 1.4%	Increased use of pornography: 24.4% Reduced masturbation: 7.7%	Sexual intercourse per week 0: 41% 1: 28.3% 2: 11.6% 3: 8.2% ≥4: 10.1%				7
Coombe,^34^ Australia	965	Men: 290 Women: 675 Heterosexual	18-29: 66.3% ≥30: 33.7%	Using sex toys alone reduced (18.3% to 8.3%) but using sex toys and masturbating more often. Sex with a girl-/boyfriend or casual hookup: reduced (45.1% to 41.8%) Dating app use: reduced generally (42.1% to 27.3%) and increased for chatting or texting (89.8% to 94.5%) and for virtual dates: (2.6% to 17.2%)	Reduced: 53.5% Masturbation: increased 6.0% Solo sex activities: increased 14.6% Sex with a spouse: increased (35.3% to 41.7%)				7
Mollaioli,^20^ Italy^c^	6821	Men: 2644 Women: 4177 Heterosexual	32.83 ± 11.24	No sexual activity during lockdown (*P* < .05) Separated from partner (*P* < .001) Upper PHQ-9 scores for women (*P* < .001) Reporting no sexual activity during lockdown (*P* < .05) Separated from partner (*P* < .001)				Upper women GAD-7 scores (*P* < .001) No significant contact among living with the partner, gender, and sexual activity during lockdown	8
Mumm,^26^ German	414	Men: 248 Women: 166 Heterosexual Homosexual: 23.4% Bisexual: 6.7%	18-69		Increased in all groups: Heterosexual men (*P* < .001) vs women (*P* < .05) Homosexual (*P* < .05) Bisexual (*P* < .001)			Masturbation increased in all groups	7
Omar,^33^ Egypt	696	Women: 479 Men: 217 Heterosexual	16-75	IIEF-5: 21.9 ± 3.2 FSFI: 21.6 ± 7.1 (decreased; *P* < .001)			Reduced (*P* < .001)		8
Panzeri,^27^ Italy	124	Women: 91 Men: 33 Heterosexual: 117 Bisexual: 5 Homosexual: 2	34.01 ± 8.71	Changes in women’s sexual function (FSFI): decreased (*P* = .001)		Cluster 1/2^d^ SDI total: 46.70/48.65 SDI dyadic: 32.12/32.96 SDI solitary: 14.58/15.69		Cluster 1/2^d^ BISF-W auto-erotism: 2.55/2.90 BISF-W couple sexuality: 3.44/3.55 PHQ-15: 5.00 ± 3.87/4.66 ± 2.89 DASS-21 total: 17.61/12.54 DASS depression: 6.18/4.27 DASS anxiety: 3.26/2.24 DASS stress: 8.18/6.03 QMI: 39.61/38.42	7
Szuster,^28^ Poland	1644	Women	25.11 ± 7.09	Total FSFI: 27.01 ± 7.61 (*P* < .001)	Reduced (*P* < .001)		Reduced (*P* < .001)		8
Yuksel,^24^ Turkey	58	Women	27.6 ± 4.4	Total FSFI: reduced (*P* = .001) Desire: increased (*P* = .011) Arousal: reduced (*P* = .001) Lubrication: increased (*P* = .503) Orgasm: reduced (*P* = .001) Pain: reduced (*P* = .121)		Increased (*P* = .011)	Reduced (*P* = .045)		7
Hensel,^30^ USA	1010	Women: 1010 Heterosexual: 91.9% Homosexual: 3.2% Bisexual: 3.4% Something else: 1.4%	34.4 ± 17.1	Decreased giving/receiving oral sex, sending/receiving sexy or naked photos with a partner, looking porn/erotica with a partner, and phone/video sex/chat (ORs, 0.58-0.75) Decrease in more protective behaviors was linked to decrease in kissing, hugging with a partner, solo masturbation, vaginal sex, partnered masturbation/genital touching, and watching porn/erotica with a partner (ORs, 1.03-1.10). Members with a greater belief in the likelihood of COVID-19 was linked to more partnered masturbation (OR, 1.09), vaginal sex (1.08), sending/receiving sexy or naked photos with a partner (1.09) in the past month.				More COVID-19 knowledge was related with lower likelihood of Increased solo masturbation, receiving oral sex, partnered masturbation/genital touching, receiving oral sex, vaginal sex and phone/video sex/chat with a partner (ORs, 0.49-0.80). More consequence perception was also related to decreased solo masturbation (OR, 1.10) and watching porn/erotica with a partner (OR, 1.07)	8
Hammoud,^35^ Australia	940	Men: 940 Gay: 871 Bisexual: 49	39.9 ± 13.4	Reduction of usual number of partners per day: 58.1% Avoidance of close physical contact and sex with casual partners: 66.1%					7
Harkness,^21^ USA^e^	20	Men: 20 Gay: 18 Bisexual: 2	32.7 ± 11.90	Issues that reflect LSMM sexual behavior during COVID-19: continued sexual activity before COVID-19, often followed by “quarantine fatigue,” reduction of the number of partners, calibration of COVID-19 prevention and sex, using sexual interacting apps in new ways.	Increase in the frequency of sexual intercourse with the primary partner				-
Hyndman,^29^ England	814	Men: 814 MSM	40.25 (33-48)	Reduction of the number of partners: 75% Changes of sexual behavior: 76% (*P* < .001)				73% had discussed COVID-19 transmission risks with their sexual partners. No emotional change following sex (46%). Isolation, loneliness (48%), and anxiety and stress (27%) triggered sexual activity.	7
Nelson,^31^ US	154	Men: 154 Gay/bisexual	16 ± 9		No sexual activity: 38% (unclear if a change due to COVID-19)			Increased virtual sex (nude photographs, sexting), 10%; increased solo masturbation, 13%; increased pornography use, 5%; being stuck at home, 5%	6
Shilo,^36^ Israel	2562	Men: 2562 MSM	37 ± 11.3	Casual sex during the pandemic: 39.5% MSM: decrease in sexual risk and limited sexual record (in specific, kissing)	Spending more time in dating applications			Being single, younger, and depressed predicted engaging in casual sex during social distancing. Increased use of phone sex, webcams, and porn.	8
Torres,^32^ Brazil	3486	Cisgender men: 97.5% Transgender men: 0.3% Transgender women: 0.8% Gender fluid: 1.3% Gay: 84.9%	32.75 (27-40)		Sex abstinence during social distancing: 45.4% Casual partners: 76.8% (mainly online) Virtual sex: 28.8%			High fear to get COVID-19 in transgender/nonbinary and moderate in cisgender. Physical, sexual, or emotional abuse was seen in transgender/nonbinary (2.2%) and in cisgender (2.3%; *P* = .96)	7

Abbreviations: BISF-W, Brief Index of Sexual Functioning for Women; DASS-21, Depression Anxiety Stress Scales–21; FSFI, Female Sexual Function Index; GAD-7, General Anxiety Disorder–7; IIEF, International Index of Erectile Function; LSMM, Latinx sexual minority men (gay, bisexual, and MSM); MSM, men who have sex with men; NOS, Newcastle-Ottawa Scale; OR, odds ratio; PHQ-9, Patient Health Questionnaire–9; QMI, Quality of Marriage Index; SDI, Sexual Desire Inventory.

a Each study was based on a cross-sectional design unless noted otherwise.

b Frequency of sexual intercourse.

c Case-control.

d Cluster1 (N = 57) is characterized by the higher number of changes in sexual life during COVID-19, Cluster 2 (N = 67), on the contrary, includes the participants who have perceived very few or no differences in sexuality. Values are presented as mean.

e Qualitative study.

### Meta-analysis

We performed a quantitative synthesis of the included studies in accordance with the search strategy. For the purpose of the meta-analysis, we used the Comprehensive Meta-analysis version 2 software. We utilized the standardized mean difference (SMD) to evaluate the effects of the COVID-19 pandemic on sexual activity, functioning, and satisfaction. In statistics, the SMD is a measure of effect size. It is the mean divided by the standard deviation of a difference between 2 random values each from 1 of 2 groups.

## Results

**Figure 1:**
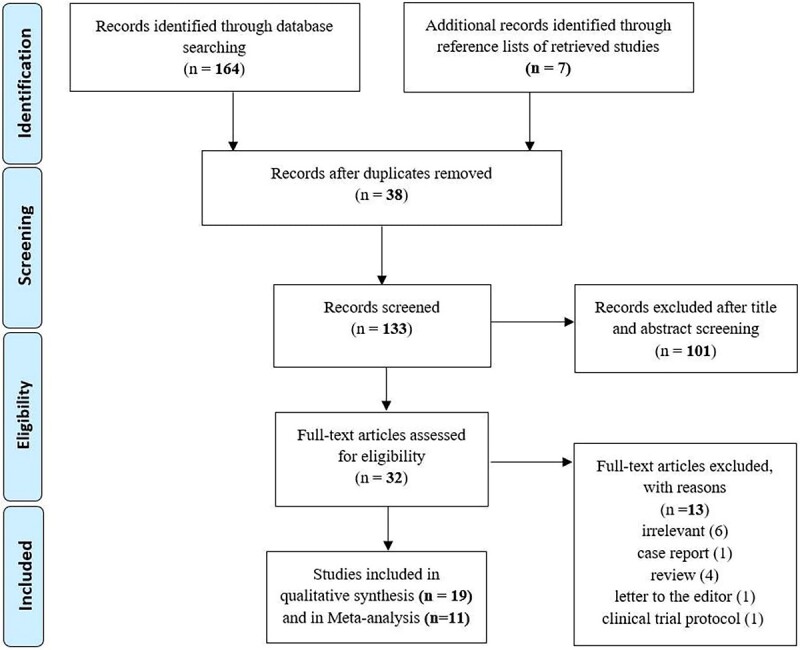
PRISMA flowchart.

In the primary search, we identified 164 studies from the searching stage and 7 from the manual search. After elimination of duplicates (38 articles), 133 remained for screening. The next step was to process studies that had an irrelevant title or abstract from the exclusion criteria. We selected 32 articles for further analysis. Finally, we included 19 studies in the qualitative synthesis and entered 11 studies in a meta-analysis, with a total of 12 350 participants. Figure 1 demonstrates the PRISMA flowchart. Two independent reviewers assessed all 19 full-text publications, and there was substantial agreement between them for excluding articles (kappa score = 0.75). All studies were cross-sectional designs except for 2: a case-control study^20^ and a qualitative study.^21^ We obtained studies from all 5 continents: 5 were conducted in Asia,^16^^,^^22-24^ 6 in Europe,^20^^,^^25-29^ 5 in America,^15^^,^^21^^,^^30-32^ 1 in Africa,^33^ and 2 in Australia.^34^^,^^35^ An overall 19 313 men, women, and minority genders in the age range of 16 to 75 years were studied in these publications. Six studies were conducted on sexual minorities and 13 on the general population.

### Sexual activity change during the COVID-19 pandemic

Eight studies investigated the frequency of sexual intercourse. Six revealed a reduction in sexual activities.^16^^,^^22^^,^^23^^,^^25^^,^^28^^,^^34^ Only Harkness et al (Latinx sexual minority men)^21^ and Mumm et al reported an increase in the frequency of intercourse in their participants (heterosexual, homosexual, and bisexual).^26^

**Figure 2:**
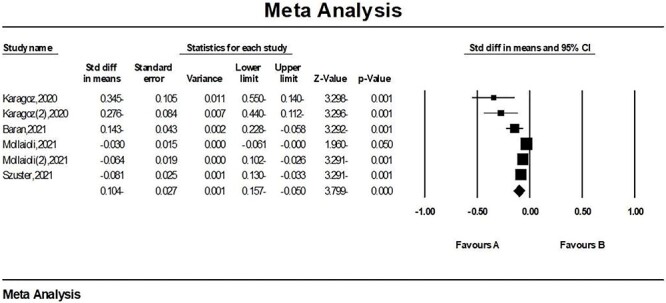
Forest Plot of Sexual Activity changes during the COVID-19 pandemic. CI, confidence interval; Std diff, standardized mean difference, SMD.

To investigate the variations of sexual activity during the COVID-19 pandemic era, a meta-analysis was conducted with a sample size of 8838 from 4 studies: SMD = −0.104 (95% CI, −0.157 to −0.050), *P* < .0001 (Figure 2). Sexual activity significantly declined during the COVID-19 pandemic era. *I*^2^ = 77.40% indicated high heterogeneity. Therefore, a random model was used for meta-analysis.

**Figure 3:**
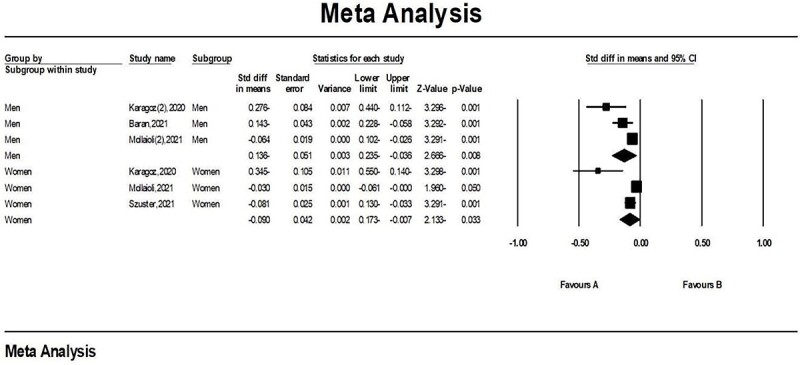
Sexual Activity and gender during the COVID-19 pandemic. CI, confidence interval; Std diff, standardized mean difference, SMD.

Due to the use of the random model, 8838 people of 4 studies were entered into the meta-analysis to investigate the sexual activity change according to gender during the COVID-19 pandemic. Subgroup analyses were conducted by gender: 5821 women, SMD = –0.090 (95% CI, 0.173 to −2.133), *P* < .033; 3017 men, SMD = −0.136 (95% CI, −0.235 to −0.036), *P* < .008. The subgroup analyses indicated that in both genders, sexual activity significantly declined during the COVID-19 pandemic (Figure 3). Moreover, the number of sexual partners decreased,^21^^,^^22^^,^^29^^,^^35^ as did the number of casual partners.^34-36^ One study reported that virtual casual partners increased.^32^

### Sexual function change during the COVID-19 pandemic

Sexual function in both genders decreased. In a long-term lockdown, the FSFI scores (Female Sexual Function Index) reported by 3 studies were significantly reduced in women.^16^^,^^23^^,^^25^^,^^33^ For men, IIEF scores (International Index of Erectile Function) were significantly reduced during the pandemic as compared with prepandemic scores.^23^ Of 5 studies reporting sexual desire changes, 4 showed reduced desire.^22^^,^^23^^,^^25^^,^^28^

**Figure 4:**
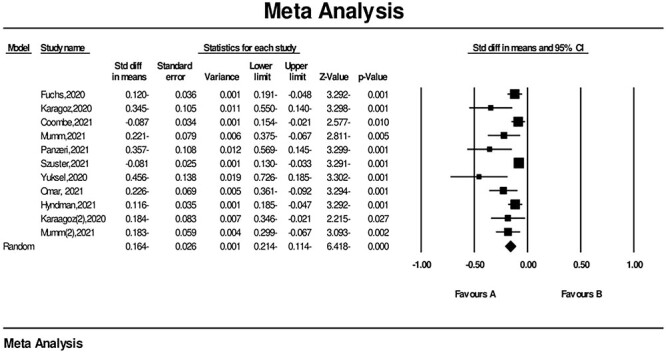
Forest plot of sexual function changes during the COVID-19 pandemic. CI, confidence interval; Std diff, standardized mean difference, SMD.

A meta-analysis with a sample size of 5401 (9 studies) showed a significant reduction in female sexual function during the COVID-19 pandemic: SMD = –0.164 (95% CI = −0.214, −0.114), *P* < .0001 (Figure 4). Moreover, *I*^2^ = 58.91%, indicated high heterogeneity. Therefore, a random model was used.

**Figure 5:**
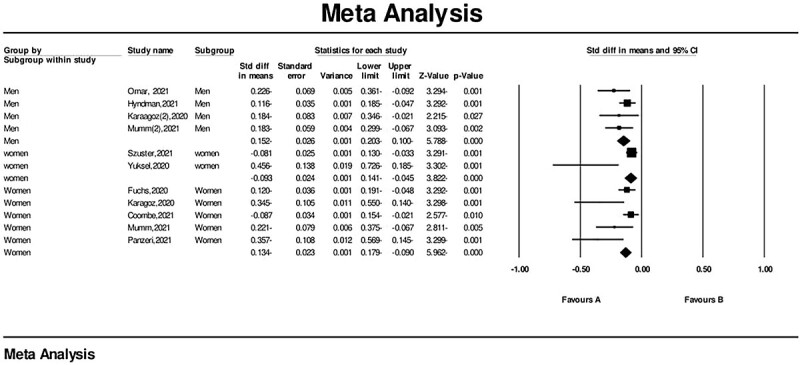
Forest plot of sexual function changes and gender during the COVID-19 pandemic. CI, confidence interval; Std diff, standardized mean difference, SMD.

The subgroup meta-analysis based on gender showed that sexual function scores significantly decreased during the COVID-19 pandemic: 3974 women, SMD = −0.134 (95% CI = −0.179 to −0.90), *P* < .001; 1427 men, SMD = −0.152 (95% CI, −0.20 to −0.100), *P* < .001) (IIEF for men and FSFI for women) (Figure 5).

### Sexual satisfaction during the COVID-19 pandemic

**Figure 6:**
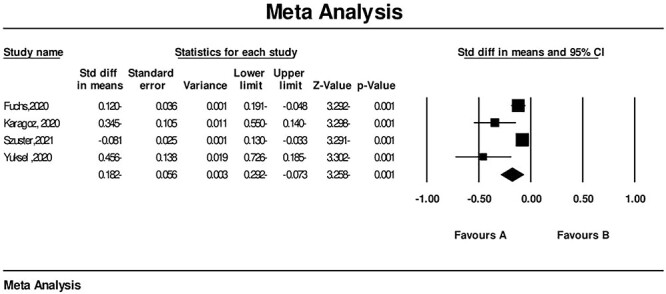
Forest plot of sexual satisfaction changes during the COVID-19 pandemic. CI, confidence interval; Std diff, standardized mean difference, SMD.

Sexual satisfaction significantly decreased during the COVID-19 pandemic according to the meta-analysis, with a sample size of 2711 from 4 studies: SMD = −0.182 (95% CI, −0.292 to −0.073), *P* < .001 (Figure 6). *I*^2^ = 76.44% indicated high heterogeneity; consequently, we used a random model for meta-analysis. Studies investigating sexual satisfaction reported that participants were less satisfied with their sexual lives.^23-25^^,^^33^

### Sexual behaviors during the COVID-19 pandemic

Individuals’ sexual behaviors changed during the pandemic. These changes in different studies could not be meta-analyzed. The participants reported using sex toys and masturbating in lockdown, more or for the first time^34^; they also washed their hands before and after sex. Greater COVID-19 knowledge was associated with lower likelihood of increased solo masturbation, partnered masturbation or genital touching, receiving oral sex, vaginal sex, and phone or video sex/chat with a partner in the past month (all odds ratios, 0.49-0.80).^30^ Also, enacting more protective behaviors was related to decreases in hugging, kissing, cuddling with a partner, solo masturbation, partnered masturbation or genital touching, vaginal sex, and watching porn or erotica with a partner (all odds ratios, 1.03-1.10).^30^

MSM, men who have sex with men, reduced their sexual risk and limited their sexual repertoire (in particular, kissing). They also spent more time in dating applications than in the pre–social distancing period and increased their use of phone sex, webcams, and porn consumption.^30^^,^^36^ Isolation and loneliness (48%) and anxiety and stress (27%) were reported to trigger sexual activity.^29^ Other participants in these studies noted an increase in their use of pornography without increasing masturbation or virtual sex (sexting, exchanging nude photographs, etc).^15^^,^^31^^,^^32^^,^^36^ Sexual desire and arousal decreased in both genders but mainly in women.^20^^,^^22-25^^,^^27^^,^^33^

### Publication bias

**Figure 7:**
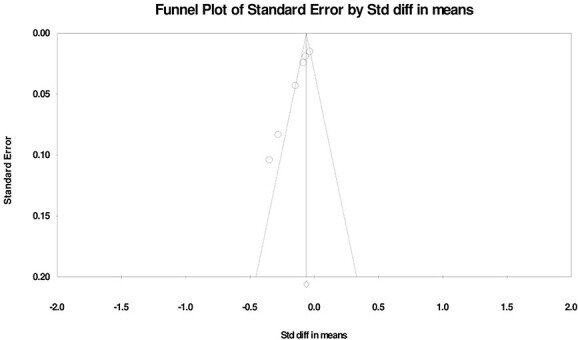
Funnel plot of changes in Sexual Activity during the COVID-19 pandemic.

**Figure 8:**
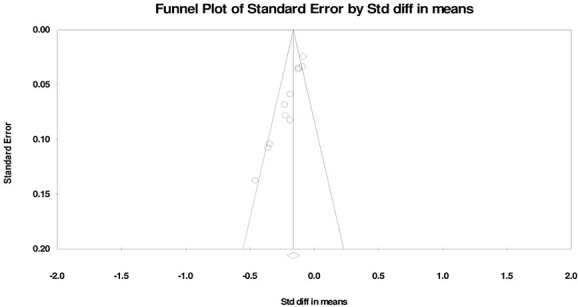
Funnel plot of changes in Sexual Function changes during the COVID-19 pandemic.

**Figure 9:**
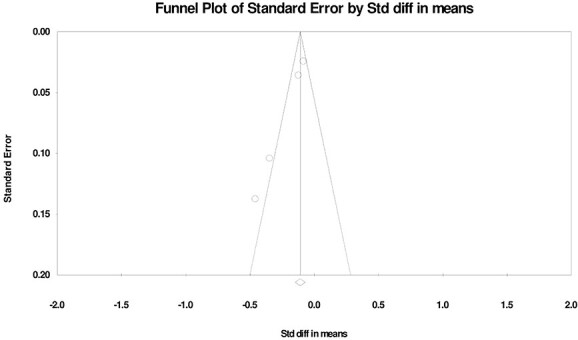
Funnel plot of changes in Sexual Satisfaction during the COVID-19 pandemic.

Publication bias was examined by means of a funnel plot: a visual tool to identify publication bias for studies on sexual activity (Figure 7), sexual function (Figure 8), and sexual satisfaction (Figure 9). The sexual activity funnel plot shows that the studies had high accuracy and low error. The sexual function funnel plot shows that most studies had high accuracy and low error, and the funnel plot of sexual satisfaction indicates that the 2 studies had low error and high accuracy.

## Discussion

This systematic review and meta-analysis analyzed the existing research evidence to try to find out if sexual function, activity, and satisfaction changed during the COVID-19 pandemic. The global response to the COVID-19 pandemic highlighted the health inequalities and the disproportionate social and economic burdens on women, girls, and vulnerable populations, which affected their health, well-being, and sexual function.^37-39^ Given that there is no definitive cure for the coronavirus, almost all health system resources were devoted to the treatment of the disease and for the supply of personal protective equipment. Accordingly, other aspects of physical and mental health and sexual health were ignored. Little is known about the impact of COVID-19 on sexual health and behaviors in a general or high-risk population. There is, however, evidence that supports the notion that COVID-19 may impair sexual health directly and indirectly.^40^^,^^41^ Given the effects of the COVID-19 pandemic on sexual and reproductive health, the results of this study will provide accurate and useful information on different aspects of sexual function and behavior during times of global health problems.

In this review, the results of some studies suggested that the frequency of sexual intercourse was reduced during the pandemic.^15^^,^^22^^,^^23^^,^^25^^,^^35^ Only 1 study reported an increase in the frequency of intercourse and masturbation.^26^ The decrease in sexual activity during the COVID-19 pandemic can be caused by the following reasons: chronic stress, women’s fear of getting pregnant, lower sexual desire, a decrease in the number of sexual partners due to social distancing, and the presence of children at home.^25^^,^^42^ Social distancing has made using phone sex and porn more common, as compared with the pre–COVID-19 pandemic period.^36^ One study did show an increase in the frequency of sexual intercourse; however, given that in a pandemic, couples may spend more time at home, they may as a result have more opportunities to have sex. Also, sexual activity as a coping strategy can reduce the stress of pandemics and quarantine.^26^^,^^41^

There is conflicting evidence whether the pandemic had an impact on sexual function among women.^43^ In line with the current review, a review with 34 articles from 18 countries suggested a deterioration of women’s sexual function across countries, with an emphasis on sexual desire.^44^

Research on the topic of gender and sexual function and satisfaction has not fully taken into account the experiences of women from developing countries. These women may face unique challenges that are not captured by studies that primarily focus on women from Western cultures. Gender inequalities may play a significant role in lower levels of sexual function and satisfaction for women and may also contribute to a wider “pleasure gap” between men and women.^44^ According to our findings, in a long-term lockdown, the FSFI score was reduced in women.^16^^,^^25^^,^^33^ Some women had significant alterations in aspects of sexual functioning and were more in need of help. One study showed that women who did not work had lower FSFI scores than women who worked during the pandemic. Lack of activity and the feeling of boredom caused by the daily routines were considered factors in reducing sexual desire.^25^ In another systematic review, working status was associated with sexual life.^43^ During pandemic restrictions, unemployment was common in many societies. Therefore, maintaining work is essential during lockdowns for individuals’ mental health and sociosexual well-being. In a study among Italian couples, the main reasons behind the changes in sexuality in women appeared to be worries, lack of privacy, and stress.^42^ In line with the current review, a review of 34 articles from 18 countries found a correlation between declines in women’s sexual function and satisfaction during the pandemic and negative mental health outcomes, such as lower psychological adjustment, higher levels of anxiety and depression, and increased stress and worry. This implies that the pandemic has had a negative impact on women’s sexual health and that this is linked to their overall mental well-being.^44^

It seems that people need higher levels of resilience, by doing, for example, exercise, yoga, or meditation, to overcome the negative aspects of lockdown and to relieve stress and worries to preserve their quality of sexual life and health. Also, preserving privacy and personal space in special conditions such as quarantine can help to further protect people’s sexual well-being.

A study revealed that more COVID-19 knowledge in the past month was related with a lower likelihood of increased solo masturbation, partnered masturbation or genital touching, receiving oral sex, vaginal sex, and phone or video sex/chat with a partner.^30^ More knowledge about COVID-19 should be accompanied by learning about protective measures, including wearing a mask while having sex with people with symptoms. During health emergencies, it is important to prioritize sexual health, as it is connected to overall well-being, and to provide recommendations on how to maintain sexual satisfaction while minimizing the risk of contamination. It may be beneficial to explore alternative ways of experiencing sexuality, such as through dating apps for chatting and virtual dates, as well as virtual sex.

The IIEF score in men significantly decreased during the pandemic as compared with the prepandemic period.^23^ Although studies show that women experienced more stress^45^^,^^46^ and depressive symptoms^47^ than men during and after the pandemic, the psychological consequences of the pandemic can reduce sexual function in men and women. In addition, chronic stress associated with higher cortisol levels can cause hormonal disorders and increase sexual dysfunction in both sexes.^48^ Hsieh et al reported that COVID-19 infection harms men’s health and erectile function by affecting their biological systems and mental health.^49^ A study from Turkey revealed that sexual avoidance and solitary sexual approach behaviors, such as masturbation or watching sexual videos, increased in men and women during the pandemic as compared with the prepandemic period.^23^ Fear of transmitting the virus to a sexual partner during the incubation period in patients with positive COVID-19 status^28^ may lead to both sexes choosing solitary sexual activity. Coombe et al^34^ found that individuals’ sexual behaviors also changed during the pandemic. The participants in these studies reported using sex toys and masturbating more often in lockdown. Being away from a sexual partner and spending a lot of time in virtual space led to the use of sex toys and masturbation in relation to sexual activity, especially in people who lived alone.^16^ This review revealed that oral sex, partnered genital touching, and vaginal sex were sexual behaviors that declined among sexual partners during this period. In addition, there was a reduction in some other sexual behaviors, such as hugging, kissing, and holding hands.^30^^,^^36^ A lack awareness of how COVID-19 infections are transmitted and the effect of contradictory and unclear news in the mass media led to fears of the possibility of transmitting the infection through close contact and changed sexual behaviors with sexual partners during the pandemic. An increase in hand washing before and after sex was also noted,^34^^,^^36^ since such hygiene measures were encouraged in lockdowns. An unwelcome result of the social changes resulting from behaviors during the COVID-19 pandemic has been the increased occurrence or worsening of obsessive-compulsive disorders.^50^ Another negative phenomenon is that loneliness can result in fewer partner-bonding behaviors and reduced partnered sexual behaviors.^30^

Participants in studies reported increased use of pornography without increasing masturbation or virtual sex (sexting, exchanging nude photographs, etc).^15^^,^^31^^,^^32^ School closures and teaching online during the pandemic gave adolescents more access to sexually explicit content in virtual space. The consequences of social distancing, such as reduced social relationships, distance from friends, and reduced physical activity, can cause mental health problems, including depression and anxiety^51^; as a result, the use of pornography and virtual sex could be recommended as a potential solution to deal with mental health disorders.^31^

Meta-analysis of 4 studies showed that sexual satisfaction significantly decreased during the COVID-19 pandemic. Studies reported that participants were less satisfied with their sexual lives.^43^ Similarly, in a cross-sectional online survey in Luxembourg, sexual satisfaction decreased as compared with before the introduction of COVID-19 restrictions, while sexual problems increased during the COVID-19 measures. Factors associated with increased odds of sexual satisfaction included having a steady relationship before COVID-19 restrictions, engaging in sexting, reporting good mental health, and not altering alcohol intake.^52^ Also, a cross-national study revealed that generally negative effects of the pandemic on close relationships varied by the type of couple (cohabitants or noncohabitants), the time interval (curfew period or not), and individual dispositions (especially attachment avoidance). Among pandemic-related stressors, mobility restrictions had a negative impact on the sexual satisfaction of noncohabitating individuals.^53^ On the contrary, in a cross-sectional study performed in Poland, no direct association was found between sexual satisfaction and levels of restrictions during the lockdown. Sexual satisfaction was significantly worse among single participants than those living as part of a couple. No gender differences were noted in sexual satisfaction.^4^

Since the COVID-19 pandemic disrupts public health, it can directly and indirectly affect sexual health. Given the importance of sexual health to overall health and well-being,^54^ health care providers and policy makers should assess individual sexual functioning and take steps to address sexual health issues during the pandemic.

This was a summary survey: The results are based on data from different countries with specific pandemic characteristics and specific cultural and social practices related to sexual health and behavior. Therefore, the results cannot be generalized to large populations involved in pandemics.

Health care providers and policy makers could use the findings of this study to help address COVID-19–related sexual health and reproductive problems in addition to supporting individuals and groups in their health and personal relationships.

## Conclusion

We have shown that the COVID-19 pandemic led to challenges and changes to individuals’ sexual behaviors. The focus for health care professions during a pandemic must be to protect all dimensions of health for the population by attempting to limit its spread, to care for individuals who acquire the infection, and to diminish adverse effects of the pandemic on mental and psychosexual health. Our review findings identified some factors that had negative effects on people’s sex lives, such as unemployment, psychological distress, lack of private space, and excessive knowledge about COVID-19. To reduce the negative impact of these factors, we suggest that efforts for preventive strategies be concentrated between pandemics, while ensuring that there is information available to the population during a pandemic for help in times of psychological distress or crisis.

### Strengths and limitations

The strengths of the review include independent duplicate data extraction by 2 reviewers, assessing the quality of the recruited studies, and conducting sensitivity analysis for identifying the high risk of biased studies. The review was enhanced by manual searching of the reference lists of the retrieved studies and by the inclusion of material from conference proceedings and as much gray literature as possible.

There are some limitations to the study. One is that since the reviewed studies were published in the English language, studies reported in other languages may have been missed and, as such, some relevant cultural differences as well. It is possible that we might have missed some relevant studies due to possible inadequacies in the study selection processes. There is also the possibility that individual judgments made by the reviewers led to instances of bias in the choice of selected articles.

## Funding

None declared.

*Conflicts of interest:* None declared.

## Ethical approval

This article does not contain any studies with human participants or animals performed by any of the authors. Institutional review board approval is not applicable, since this study is a systematic review and secondary analysis.

## Supplementary Material

Clean_abstract_qfad005
