# Correction to: Changes in sexual activities, function, and satisfaction during the COVID-19 pandemic era: a systematic review and meta-analysis

**DOI:** 10.1093/sexmed/qfad027

**Published:** 2023-05-08

This is a correction to: Kowsar Qaderi, Mansoureh Yazdkhasti, Sanaz Zangeneh, PhD, Bahar Morshed Behbahani, Mehri Kalhor, Ahmadreza Shamsabadi, Younes Jesmani, MD, Solmaz Norouzi, PhD, Mehrnaz Kajbafvala, Rasa Khodavirdilou, PhD, Nahid Rahmani, Masoumeh Namadian, Sajjad Ghane Ezabadi, MD, Ibrahim Alkatout, Esmaeel Mehraeen, Dara Rasoal, PhD, Changes in sexual activities, function, and satisfaction during the COVID-19 pandemic era: a systematic review and meta-analysis, *Sexual Medicine*, Volume 11, Issue 2, April 2023, qfad005, https://doi.org/10.1093/sexmed/qfad005

In the originally published version of this manuscript, Esmaeil Mehraeen's name and affiliation were incorrectly presented. The correct information for this author is:

Esmaeil Mehraeen

Department of Health Information Technology, Khalkhal University of Medical Sciences, Khalkhal, Iran.

This error has been corrected online.

