# Supplementary material for: Changes in sexual activities, function, and satisfaction during the COVID-19 pandemic era: a systematic review and meta-analysis
Source: Sex Med. 2023 Mar 24;11(2):qfad005. doi: 10.1093/sexmed/qfad005 (PMC10037164; doi:10.1093/sexmed/qfad005)
Supplement: Clean_abstract_qfad005 [file clean_abstract_qfad005.docx]

**Abstract**

**Introduction:** Little is known about the impact of coronavirus on sexual behavior, function and satisfaction.

**Aim:** The aim of the present study was to systematically review people’s sexual function and behaviors and changes in sexual activities during the COVID-19 pandemic.

**Methods**: Comprehensive searches in PubMed, Web of Science, and Scopus were conducted using keywords in accordance with MeSH terms, including COVID-19, SARS-CoV-2, Corona virus, sexual health, sexual function, sexual dysfunctions, sexuality, sexual orientation, sexual activities, and premarital sex. Two reviewers independently assessed full-text articles according to predefined criteria (original design, English studies, and investigating either the general population or sexual minorities).

**Results:** Risk of bias in the studies was assessed by Newcastle-Ottawa Scale (NOS), and data pooled using random-effects meta-analyses. We utilized the Standardized Mean Difference (SMD) to evaluate the effects of the COVID-19 pandemic on sexual activity, functioning and satisfaction. We included 19 studies in the analysis, and 11 studies in the meta-analysis, with a sample size of 12,350. To investigate the “sexual activity” changes, a sample size of 8838 was entered in the sub-group analysis that showed a significant decrease in both genders (n=5821 women, p <0.033), and (n=3017 men, P<0.008). A sub-group meta-analysis showed the “sexual function” of men and women during the COVID-19 pandemic significantly declined (3974 women, p< 0.001); and n=1427 men, p< 0.001). Sexual desire and arousal decreased in both genders, though mainly in women. In investigating “sexual satisfaction” changes during the COVID-19 pandemic, a meta-analysis with a sample size of 2711 showed a significant decrease (p <0.001). The most indicative changes in sexual behaviors during the pandemic were the increase in masturbating and sex toys usage. Greater COVID-19 knowledge was associated with lower masturbation, oral sex, and vaginal sex. The more protective behaviors were associated with less hugging, kissing, cuddling, genital touching, watching porn with a partner, and vaginal sex.

**Conclusion:**  The COVID-19 pandemic led to increased challenges and changes for individuals’ sexual behaviours. Efforts for preventive strategies should therefore be concentrated between pandemics, whilst also ensuring that there is information available to the population during a pandemic for help in times of psychological distress or crisis.
